# An investigation of the risk factors of chronic obstructive pulmonary disease in natural population-based cohorts in China – a nested case-control study

**DOI:** 10.3389/fpubh.2023.1303097

**Published:** 2023-12-08

**Authors:** Yixin Xu, Hongjun Zhao, Chunchun Yu, Yuqian Wang, Hao Xu, Zhe Weng, Chengshui Chen, Haizhou Mao

**Affiliations:** ^1^Key Laboratory of Interventional Pulmonology of Zhejiang Province, Department of Pulmonary and Critical Care Medicine, The First Affiliated Hospital of Wenzhou Medical University, Wenzhou, China; ^2^Department of Pulmonary and Critical Care Medicine, The Quzhou Affiliated Hospital of Wenzhou Medical University, Quzhou People’s Hospital, Quzhou, China; ^3^Department of Mathematics, Zhejiang Industry and Trade Vocational College, Wenzhou, Zhejiang, China

**Keywords:** chronic obstructive pulmonary disease, exposures, symptoms, previous history, analyze

## Abstract

**Background:**

Chronic obstructive pulmonary disease (COPD) has become one of the most significant chronic diseases in China. According to conventional wisdom, smoking is the pathogenic factor. However, current research indicates that the pathophysiology of COPD may be associated with prior respiratory system events (e.g., childhood hospitalization for pneumonia, chronic bronchitis) and environmental exposure (e.g., dust from workplace, indoor combustion particles). Dyspnea, persistent wheezing, and other respiratory symptoms further point to the need for pulmonary function tests in this population. Reducing the burden of chronic diseases in China requires a thorough understanding of the various factors that influence the occurrence of COPD.

**Methods:**

Using a cohort from the natural population, this study used nested case-control analysis. We carried out a number of researches, including questionnaire surveys and pulmonary function testing, in the Northwest and Southeast cohorts of China between 2014 and 2021. After removing any variations in the baseline data between patients and control subjects using propensity score matching analysis, the risk factors were examined using univariate or multivariate regression.

**Result:**

It was discovered that prior history of chronic bronchitis, long-term wheezing symptoms, and environmental exposure—including smoking and biofuel combustion—were risk factors for COPD. Dyspnea, symptoms of mobility limitation, organic matter, and a history of hospitalization for pneumonia at an early age were not significant in the clinical model but their incidence in COPD group is higher than that in healthy population.

**Discussion:**

COPD screening effectiveness can be increased by looking for individuals with chronic respiratory symptoms. Smokers should give up as soon as they can, and families that have been exposed to biofuels for a long time should convert to clean energy or upgrade their ventilation. Individuals who have previously been diagnosed with emphysema and chronic bronchitis ought to be extra mindful of the prevention or advancement of COPD.

## Introduction

The significant death rate linked with chronic obstructive pulmonary disease (COPD) has made it a global public health concern. By 2030, it is predicted to rank as the third most common cause of death globally (1, 2). A survey on the prevalence of COPD in China was carried out by Zhong et al. between 2002 and 2004 In China, the overall COPD prevalence was 8.2% (males: 12.4%; females: 5.1%) (3). Ten years later, the Chinese Lung Health (CPH) survey by Wang et al. revealed that 8.6% (95% CI7.5–9.9) of Chinese people had COPD overall in 2012–2015 (4). It is anticipated that China’s COPD burden would continue to increase significantly (5).

Small airway disease and lung parenchymal damage work together to develop COPD. Chronic inflammation brought on by a variety of conditions results in lung parenchymal damage, small airway stenosis, structural abnormalities, and impaired mucociliary function (6). Smoking cessation should be the first priority in the treatment due to it is one of the main risk factors for COPD (7, 8). Hazardous gases, dust at work and indoor air pollution are all regarded as environmental exposures that require attention. Fuel exposure and occupational exposure are probably the second most important risk factors for COPD after smoking in developing and developed countries, respectively (9, 10). COPD risk can be decreased by adding exhaust fans or upgrading biomass burners (11). Workers who have been exposed to a range of dangerous compounds have demonstrated a greater incidence of COPD and a corresponding rise in death (10, 12). A few prior conditions, particularly those pertaining to the respiratory symptoms, may potentially serve as early indicators of COPD (4). Additional risk variables for COPD included age, gender, and a low body mass index (3). The aforementioned impacting elements will be covered in this paper.

We discovered that the majority of COPD researches had been regionally oriented since Zhong et al.’s (3) survey on the total prevalence of COPD in China and Wang et al.’s (4) study. Nonetheless, China has an unequal demographic and economic distribution, which could have an effect on how the risk factors for COPD are determined. In order to conduct a retrospective inquiry and analysis, this study chose the population cohorts from 2014 to 2021 in the Northwest and Southeast of China, respectively, and then qualifying people were chosen to be included in the study. Moreover, we did not restrict our investigation to exposure to dust or gasoline. The study refined categories of exposure and included traceability of previous clinical conditions, which were not available in the two previous population-based national cohort studies.

## Methods

### Study design and subjects

A natural population cohort study served as the foundation for our cross-sectional survey investigation. Due to the significant economic and social difference between the Southeast and Northwest regions of China, the study was first separated into two lines. Zhejiang Province was picked to represent Southeastern China, and the province of Gansu was chosen to represent Northwestern China. In the second round, software was utilized to generate random numbers, which were used to select three districts or counties in each province. Third, depending on differences in urban and rural development, equal share of urban streets or rural towns were randomly selected from the designated urban or county. Lastly, based on population size, cluster units made up of villages or urban settlements were chosen with using a random cluster sampling technique. Tests of pulmonary function and questionnaire surveys were conducted among individuals who were 40 years of age or older. After the two provinces’ natural population cohorts were established, specific groups were screened for the case–control research.

The institutional review boards of the participating centers in each province approved the study’s protocol and procedures.

### Data collection

The goal of the study was explained to the subjects, and their informed consent was acquired. Every tester employed identical instruments, protocols, and questionnaires. Prior to conducting the survey, operators and interviewers underwent rigorous training. We obtained data from the research participants through a combination of in-person interviews, comprehensive physical examinations, and laboratory testing. A standardized and structured questionnaire was used by interviewers with training. The worldwide BOLD study was followed in developing the questionnaire’s content (13). Certain things were added or removed based on the social and economic conditions in two different provinces. Demographic, socioeconomic, lifestyle, diet, employment history, occupational exposure, self-reported medical history, and other details were included in the information. The definition of exposure to relevant substances was indicated in the questionnaire. For example, A smoker was classified as someone who had smoked for more than 6 months straight. Occupational exposure was defined as more than a year of exposure to chemicals or dust at work (14). The discussion section goes over other pertinent definitions. Exclusions included recent surgery, history of stroke, pregnancy, and other conditions that would influence the pulmonary function test (15). Following the determination of spirometry eligibility, the individuals were scheduled for spirometry evaluation.

### Diagnostic criteria

Although some studies have suggested that the LLN (lower limit of normal) is more valuable for the diagnosis of COPD, we adhere to the GOLD criteria: COPD was defined as FEV1/ FVC < 70% (FEV1: Forced Expiratory Volume in the first second; FVC: Forced Vital Capacity; The ratio serves as an indicator of the extent of airway obstruction) after the bronchodilator test (inhalation of salbutamol 400 μg for at least 15 min) (16, 17). All populations using uniform spirometeres. All subjects underwent pulmonary function tests twice. If the post-bronchodilator FEV1/FVC was less than 0.70 during the two tests, we confirmed it on a separate occasion by repeating lung function test. This is because of the inaccuracy of making a diagnosis of COPD based on a single lung function measurement (18). Bronchodilators were prohibited for 48 h prior to testing.

### Data processing

Chi-square tests were used to ascertain differences across variables, and exposure rates were computed as crude rates and 95% confidence intervals. Using univariate/multivariate regression, the odd ratio (OR) and 95% confidence interval (CI) of COPD and possible risk variables were determined. SPSS version 27 and SAS version 9.4 were used for data analysis. Based on two-sided tests with a significant level of 0.05, all stated *p* values have been calculated.

## Results

### Participants characteristics

**Figure 1:**
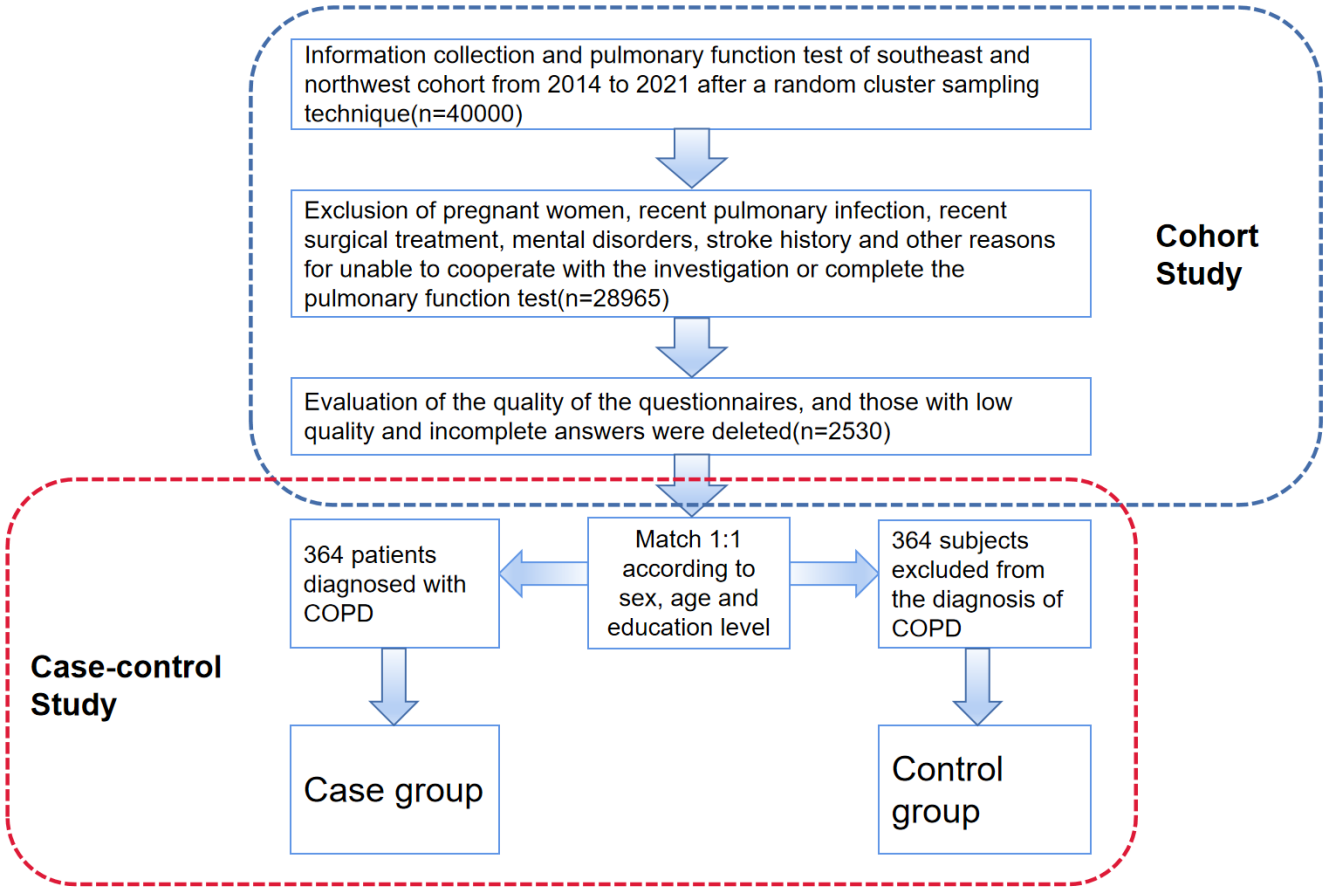
Procedures for data acquisition, screening and inclusion of subjects. The nested case-control study’s technical path is depicted in this figure. The original cohort’s formation is displayed in the upper portion of the image, along with the inclusion and exclusion criteria. The PSM method and the portion of the case-control research are displayed in the lower part of the figure.

Included in the initial natural population cohort were more than 40,000 people. 2,530 people meet every requirement included in the analysis, as seen in Figure 1. Following 1:1 propensity score matching (PSM) based on age, gender, and educational attainment between the case group and the control group, 728 patients were ultimately chosen for the nested case-control investigation.

Between the two groups, the average age was 60.74 (SD = 9.22). Men made up 75.3% of the population, far more than women. The majority of them only completed middle school or less, so the population under study has a low average level of education. The data presented in Table 1 indicates that there is no statistically significant variation in gender, age, or cultural level among the various groups.

**Table 1:** The basic demography characteristics of inclusion objects.

Characteristics	Total (*n* = 728)	Cases (*n* = 364)	Controls (*n* = 364)	*p*-value^b^
Sex				0.39
Male	548	279	269	
Female	180	85	95	
Age (years)^a^	60.74 ± 9.22	60.68 ± 9.10	60.80 ± 9.35	0.85
Education				0.73
Less than primary	348	175	173	
Middle or high school	363	179	184	
College or above	17	10	7	

a Mean (standard deviation, SD).

b Case group vs. control group.

### Clinical signs and symptoms

The rate at which symptoms emerge varies significantly between the COPD group and the healthy population. According to Table 2, the case group experienced greater respiratory symptoms than the control group. Long-term cough and phlegm did not differ between the two groups, but the case group experienced significantly greater rates of panting (28.3 vs. 15.9%), dyspnea during activities (44.2 vs. 28.0%), and limited mobility due to breathing difficulties (12.4 vs. 6.6%; *p* < 0.05).

**Table 2:** Different clinical symptoms of 2 groups.

Symptoms	Cases *n* (%)	Controls *n* (%)	*p*-value^a^	OR (95%CI)
Cough				
Yes	94 (25.8)	90 (24.7)	0.73	1.06 (0.76,1.48)
No	270 (71.2)	274 (75.3)		1.00 (reference)
Expectoration				
Yes	93 (25.5)	76 (20.9)	0.14	1.30 (0.92–1.84)
No	271 (74.5)	288 (79.1)		1.00 (reference)
Pant				
Yes	103 (28.3)	58 (15.9)	< 0.05	2.08 (1.45–2.99)
No	261 (71.7)	306 (84.1)		1.00 (reference)
Dyspnea				
Yes	161 (44.2)	102 (28.0)	< 0.05	2.04 (1.50–2.77)
No	203 (55.8)	262 (72.0)		1.00 (reference)
Activity limitation				
Yes	45 (12.4)	24 (6.6)	< 0.05	2.00 (1.19–3.36)
No	319 (87.6)	340 (93.4)		1.00 (reference)

CI, confidence interval.

a Case group vs. control group.

### Exposure factors

The respondents’ prior exposure history was charted in Table 3. According to the majority of published research, smoking was discovered to be a risk factor for COPD. Both the case and control groups had more than half of their participants smoking (both past and present); the case group even reached 64.0%, which was statistically different from the control group. The case group had less secondhand smoke (SHS) exposure than the control group, but the difference was not statistically significant.

**Table 3:** Exposure factors comparison between 2 groups.

Exposures	Cases *n* (%)	Controls *n* (%)	*p*-value^b^	OR (95%CI)
Smoking				
Yes	233 (64.0)	199 (54.7)	<0.05	1.48 (1.10–1.99)
No	131 (36.0)	165 (45.3)		1.00 (reference)
SHS^a^				
Yes	176 (48.4)	189 (51.9)	0.34	0.87 (0.65–1.16)
No	188 (51.6)	175 (48.1)		1.00 (reference)
Fuel exposure				
Biofuels	203 (55.8)	177 (48.6)	<0.05	1.33 (1.00–1.78)
Coal fuel	98 (26.9)	92 (25.3)	0.61	1.09 (0.78–1.52)
None	131 (36.0)	176 (48.4)		1.00 (reference)
Winter heating				
Yes	237 (64.3)	246 (67.6)	0.48	0.90 (0.66–1.22)
No	127 (34.9)	118 (32.4)		1.00 (reference)
Dust exposure				
Chemical	42 (11.5)	51 (14.0)	0.32	0.80 (0.52–1.24)
Metal	12 (3.3)	8 (2.2)	0.37	1.52 (0.61–3.76)
Inorganic minerals	30 (8.2)	29 (8.0)	0.89	1.04 (0.61–1.77)
Organic matter	24 (6.6)	11 (3.0)	<0.05	2.27 (1.09–4.70)
Crops	94 (25.8)	90 (24.7)	0.73	1.06 (0.76–1.48)
None	221 (60.7)	230 (63.2)		1.00 (reference)

CI, confidence interval.

a Secondhand smoke.

b Case group vs. control group.

We separated the fuels into biofuels (straw and animal dung) and coal (kerosene and coal) based on the economic and environmental conditions of the two sites. We also divided the occupational exposure into five categories: metal dust, inorganic dust (silica, coal mining, cement manufacturing, etc.), chemical dust (detergent, hair dye, smoke, etc.), organic dust (poultry feathers or other animal hair), and crop dust (planting soil, grain dust, cotton dust, etc.). The case group’s rate of long-term biofuel use was greater than that of the other group’s (55.8 vs. 48.6%) in terms of solid particle matter exposure. Although there were comparable numbers of coal users in the two groups, the case group’s rate was generally higher. In terms of occupational dust exposure, the case group was slightly more than the control group, and the two groups’ exposure amounts to various chemicals were comparable. Only the exposure to organic compounds (6.6 vs. 3.0%) showed differences.

### Previous history

The participant’s past medical history, including a few common chronic conditions, was included in our questionnaire (Table 4). In the case group, the percentage of subjects who had been hospitalized for pneumonia in childhood (6.6 vs. 3.0%), or who had been diagnosed with chronic bronchitis (18.4 vs. 8.8%) or emphysema (8.0 vs. 0.3%), was significantly higher than that of the control group, according to the comparison of prior diseases. The prior history of bronchial asthma, which has diagnostic criteria comparable to COPD but is significantly reversible with bronchodilators, did not, however, show a statistically significant difference. Additionally, there was no change in the factor—a history of tuberculosis. Furthermore, chronic diseases like diabetes and cardiovascular disease were excluded from the clinical model since there was no evidence linking them to the illness.

**Table 4:** Comparison of past disease between 2 groups.

Past history	Cases *n* (%)	Controls *n* (%)	*p*-value^a^	OR (95%CI)
Hospitalization for pneumonia in childhood				
Yes	24 (6.6)	11 (3.0)	<0.05	1.27 (1.09–4.70)
No	340 (93.4)	353 (97.0)		1.00 (reference)
Asthma				
Yes	25 (6.9)	17 (4.7)	0.21	1.51 (0.80–2.84)
No	339 (93.1)	347 (95.3)		1.00 (reference)
Chronic bronchitis				
Yes	67 (18.4)	32 (8.8)	<0.05	2.34 (1.49–3.67)
No	297 (81.6)	332 (91.2)		1.00 (reference)
Emphysema				
Yes	29 (8.0)	1 (0.3)	<0.05	31.42 (4.26–231.96)
No	335 (92.0)	363 (99.7)		1.00 (reference)
Allergic rhinitis				
Yes	6 (1.6)	10 (2.7)	0.32	0.59 (0.21–1.65)
No	358 (98.4)	354 (97.3)		1.00 (reference)
Pulmonary tuberculosis				
Yes	5 (1.4)	3 (0.8)	0.48	1.68 (0.40–7.07)
No	359 (98.6)	361 (99.2)		1.00 (reference)
Hypertension				
Yes	76 (20.9)	88 (24.2)	0.29	0.83 (0.58–1.17)
No	288 (79.1)	276 (75.8)		1.00 (reference)
Coronary heart disease				
Yes	6 (1.6)	8 (2.2)	0.59	0.75 (0.26–2.17)
No	358 (98.4)	356 (97.8)		1.00 (reference)
Diabetes				
Yes	19 (5.2)	17 (4.7)	0.73	1.12 (0.58–2.20)
No	345 (94.8)	347 (95.3)		1.00 (reference)

CI, confidence interval.

a Case group vs. control group.

### Establish clinical model

**Figure 2:**
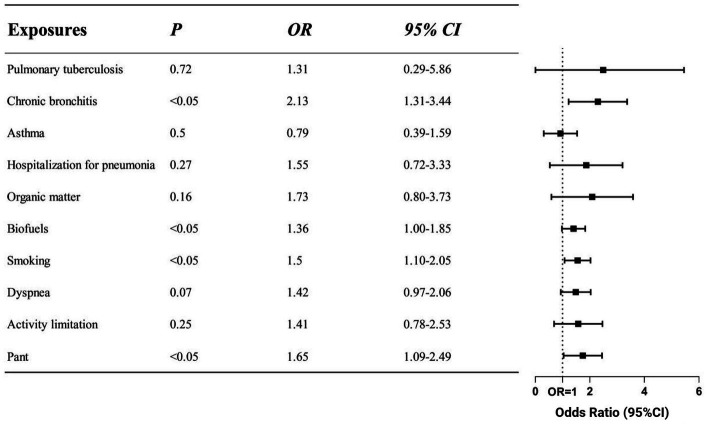
Odds ratios of different risk factors in clinical model. The screened exposure factors are displayed on the left side of the figure, while the corresponding OR values and 95%CI are displayed on the right side. The clinical model was adjusted for gender, age and education. Indicators that were positive in the comparison of respiratory symptoms, exposure factors and history of past illness were included. According to the findings, the clinical model of COPD includes smoking, using biofuel, having a history of bronchitis, and having pant symptoms as risk factors (*p* < 0.05).

While multi-factor analysis can adjust for the influence of multiple confounding factors and change the study’s findings, univariate analysis frequently yields results that are not very dependable. Figure 2’s risk factors that showed statistical significance in univariate regression were examined using logistic regression, and our clinical model contained variables that showed statistical significance in multivariate regression. Using references to pertinent literature, the clinical model also incorporated the respiratory system’s past history. Despite the fact that the univariate regression showed a substantial correlation (OR = 31.42) between the onset of the disease and a prior history of emphysema, we did not incorporate this into the clinical model due to the small number of patients with emphysema in the control group. There were worries that adding it would cause the result to be inaccurate. In the clinical model following multivariate regression, the symptoms of wheezing (OR = 1.65), smoking history (OR = 1.50), exposure to biofuels (OR = 1.36), and prior history of bronchitis (OR = 2.13) were identified as risk factors for COPD.

## Discussion

COPD is a type of chronic lung disease. In order to increase screening efficiency, a well-designed COPD screening questionnaire revealed that respiratory symptoms like cough, expectoration, dyspnea, and wheezing could be useful in identifying individuals who would benefit most from pulmonary function testing (19). The percentage of individuals in both groups who had expectoration and cough was comparable. On the other hand, the case group experienced almost twice as many symptoms of wheezing or activity limitation as the control group. Dyspnea affected a notably higher number of individuals in the case group. Using a symptom-based questionnaire in the community can increase the effectiveness of COPD screening (20). It is recommended that individuals exhibiting respiratory symptoms, particularly those with persistent wheezing, should promptly undergo a pulmonary function test to ascertain the presence of COPD. Those with respiratory problems should receive extra consideration when being screened. The COPD disease burden can be decreased with early diagnosis (21).

Smoking was found to be a significant risk factor for COPD by both Ma et al. (OR value = 1.51) and Zhong et al. (OR value between 1.27 and 1.72) (2, 3). They were all rather close to the 1.50 OR value found in our investigation. Without a question, the most important factor in COPD is smoking. It has the potential to hasten FEV1 decrease. Accelerated airway oxidative stress, airway collapse, and inadequate lung tissue repair are some of the pathophysiological processes (22). The aetiology of COPD has been steadily revealed in recent years to involve occupational exposure to substances other than tobacco smoke and air pollution (2, 23). Our research, however, does not support the conception that exposure to secondhand smoke (SHS) increases the risk of developing COPD. Our questionnaire did not specify the precision of exposure time and degree, but we hypothesize that only a certain amount of secondhand smoking exposure can cause COPD (24). Patients may experience greater success quitting smoking if their respiratory symptoms are more acute. It may be more successful to implement interventions to improve COPD patients’ adherence to smoking cessation therapy (8). It makes sense to counsel smokers to give up their habit (2, 25), not only to slow down the deterioration of their lungs but also to lessen the amount of secondhand smoke that they are exposed to in the community.

In addition to tobacco use, solid fuel use is a significant risk factor for COPD, particularly in developing nations (26, 27). The combustion of these solid fuels in domestic inefficient household stoves is the main source of indoor air pollution and have a negative impact on the respiratory system (11, 28). In Xuanwei, Yunnan Province, lung cancer and COPD have been discovered to be directly linked to the burning of these fuels (10, 29). According to the aforementioned extensive epidemiological study of COPD in China conducted by Zhong et al., exposure to indoor biomass for heating or cooking was linked to COPD (OR = 1.35 95%CI:1.20–1.52). In comparison to clean fuels, coal had an HR of 1.16 (95%CI, 1.04–1.29) and wood had an HR of 1.21 (95%CI, 1.09–1.35) for heating, according to a large prospective domestic study (30). The use of coal fuel did not significantly differ between individuals with and without COPD, according to our research. However, compared to the control group, COPD patients were more likely to use biomass fuel. After the multivariate analysis, the OR value of biomass fuel exposure was 1.36 (95%CI, 1.00–1.85), which was comparable to the findings of Zhong et al. We consider the following factors could be at play: In rural China, coal and biofuels are the primary sources of heat and cooking for almost half the population (31). About 10% of the energy used by rural homes comes from coal, while about 80% comes from biomass (32). The majority of the responders are from rural areas in northwest China. Southeast China, on the other hand, has strict control of coal due to the increased popularity of clean fuels like natural gas. The amount of coal used nationwide is lower than the amount of biofuels. Delaying the onset of COPD can be achieved by weaning off of solid fuel exposure. As a result, some members of the exposed population who burned coal fuel did not go on to get COPD. Thus, we recommend that the government tighten regulations on the use of coal and biofuels while also promoting the use of clean fuels. In order to lower PIC (products of incomplete combustion) emissions, homeowners should be encouraged to install ventilation equipment and upgrade their stoves. This is especially important for residents living in rural regions (9, 29, 32).

Apart from smoking, occupational dust exposure may have a major role in pathophysiology of COPD in developed countries (10). Proteases can be released, oxidative stress reactions can be triggered, and epithelial cells can be harmed by the accumulation of occupationally hazardous particles in the respiratory tract. These particles include inorganic dust, soot, metals, and irritants (33). During agricultural activities, the inflammatory response in the airways may be caused by microbial components in organic dust (34). According to the Jinchang cohort research in Northwest China, the adjusted OR values for the metal exposure groups with moderate and high exposure were 1.22 (95%CI, 0.85–1.76) and 1.50 (95%CI, 1.03–2.18), respectively. Like the Jinchang cohort, a sizable number of our cohort’s members were from northwest China, but the Jinchang cohort’s members were regularly exposed to heavy metals (2). Like the Jinchang cohort, a sizable number of our cohort’s members were from northwest China, but the Jinchang cohort’s members were regularly exposed to heavy metals. The goal of our research is to determine which occupational exposure affects the pathophysiology of COPD. However, in univariate and multivariate analysis, exposure to organic dust was the only factor that was significant. It has been determined that allergens, microbes, and disinfectants exposed to animal feed are risk factors for COPD (35). We did not detect any appreciable changes in the exposure rates of inorganic compounds and metal dust between the two groups, despite the fact that we thoroughly described the meaning of exposure and the common types of various substances during the questionnaire-filling procedure. The confirmation of occupational exposure in many studies, including ours, was based on self-reports. The subjects’ educational background (the majority were middle school students and lower), lack of environmental awareness, and propensity for subjective feedback may have contributed to the bias. But subjective effects such as those generated by self-report cannot be eliminated. Furthermore, rather than being exposed to a single substance at work, the majority of workers are exposed to diverse pollutants, and various kinds of pollutants may interact (36). Although the relevant evidence and OR values of different occupational exposures need to be investigated, the impact of workplace exposures on the pathogenesis of COPD should be paid attention to in any case (10).

A prior history of lung disease may increase the likelihood of developing COPD. Our research shown that in clinical regression model, a prior diagnosis of chronic bronchitis was a risk factor for COPD. Patients with chronic bronchitis and COPD have a higher chance of dying and a more severe decrease in lung function (37). According to certain research, chronic bronchitis, emphysema and asthma are three phenotypes of COPD (33, 38). In the US, the COPD Gene Study examined 10,192 adult smokers and discovered a correlation between childhood pneumonia and COPD (OR 1.40; 95%CI 1.17–1.66) (25). The univariate odds ratio was comparable to our study’s, despite the clinical model’s lack of significance for children pneumonia. This implies that early-life respiratory illness may have an impact on the development of COPD (39). Our research, however, does not point to a connection between COPD and pulmonary tuberculosis. Since tuberculosis is a treatable illness, we consider that the cause is recall bias—some people may not be aware of their prior infection history. Its impact on lung illnesses may be because to the oxidative stress and inflammation brought on by hyperglycemia, which can cause damage to the pulmonary arteries (40). However, there is now little direct evidence of mechanism linking one disease to the other’s advancement (41, 42). In a similar vein, it’s well accepted that COPD and cardiovascular illness are tightly associated (43). Common risk factors for cardiovascular disease and COPD include smoking and tobacco use (44). On the other hand, the aforementioned variables could cause cardiovascular and pulmonary illnesses to appear simultaneously. It is still up to us to discover and verify the mechanism (43, 45).

This investigation has some shortcomings. Due to the respondents’ cultural background and other limitations, the inquiry contains some recall bias, which could cause a partial variation in the results. The inclusion factors in the clinical model are still up for debate, and there may be potential elements that influence the outcome. Due to missing data from the survey and our use of PSM for data processing, fewer individuals were included in the two groups even though our overall population cohort size exceeded 40,000. We employ strict inclusion criteria in the hopes that the results from the interference of bias, but the objects may be too little.

## Data availability statement

The raw data supporting the conclusions of this article will be made available by the authors, without undue reservation.

## Ethics statement

Written informed consent was obtained from the individual(s) for the publication of any potentially identifiable images or data included in this article.

## Author contributions

YX: Writing – original draft, Writing – review & editing. HZ: Writing – original draft, Writing – review & editing. CY: Writing – original draft. YW: Writing – original draft. HX: Writing – original draft. ZW: Writing – original draft. CC: Writing – review & editing. HM: Writing – review & editing.
